# Seroprevalence of IgG Antibodies against *Echinococcus granulosus* in the Population of the Region of Thessaly, Central Greece

**DOI:** 10.1371/journal.pone.0037112

**Published:** 2012-05-17

**Authors:** Vasilios Fotiou, Eleni Malissiova, Anastasios Minas, Efthimia Petinaki, Christos Hadjichristodoulou

**Affiliations:** 1 Department of Hygiene and Epidemiology, Faculty of Medicine, University of Thessaly, Larissa, Greece; 2 Department of Medical Laboratories, Technological Educational Institute of Larissa, Larissa, Greece; 3 Department of Microbiology, General University Hospital of Larissa, Larissa, Greece; Aga Khan University, Pakistan

## Abstract

Echinococcosis notification rate in Greece, based on the most recent data, is below 0.25 per 100,000 population. To further investigate the epidemiology of echinococcosis in Greece a study was designed to determine the seroprevalence of *Echinococcus granulosus* antibodies in the population of Thessaly, Central Greece. Five hundred and forty two left over blood samples in Thessaly laboratories, were collected using a stratified convenient sampling procedure. Samples were analyzed with enzyme linked immunosorbent assay. The seropositivity found was 1.1%(95% C.I., 0.5–2.5), with 5 out of 6 seropositive results to be in the age group of over 65 (OR = 17.95, 95%CI 2.04–157.11, p value 0.009). Rural residence was also found as a risk factor to seropositivity (RR = 7.60, 95% CI 0.89–64.64, p value 0.039). Surveillance data and our study results converge that echinococcosis is being reduced in Greece, with older population to be affected mostly. These might be due to the disease transmission restriction, by the control measures being implemented. Efforts should be continued, in both animals and humans side, by increasing training campaigns and public awareness.

## Introduction

*Echinococcus granulosus* has a worldwide distribution and is responsible for 95% of echinococcosis cases in humans worldwide. The *Echinococcus*' main intermediate host is the sheep and the parasite has as point of localization, the liver and the lungs. The practice of dogs being fed with offal from ruminant livestock originating from illegal slaughtering is the main mode of transmission of *Echinococcus*. Humans are infected with contaminated food or water with dog faeces. Cases of human infection have been reported in the age from 1 to 75 years. Rural residence has been reported as an associated factor to echinococcosis. Infection rates do not differ between sexes, while 60% of the infected population remains asymptomatic forever [1], [2].

Echinococcosis incidence has been dramatically reduced in Greece but is not eradicated. Echinococcosis notification rate in Greece, based on the most recent data, is below 0.25 per 100,000 population [3]. Surveillance data, as reported by the competent authorities for the decade 1998–2009, reveal that the cases in Thessaly were 16 in total, with the latest case to be reported in 2003. According to these data, 8 cases were over 50 years old, 3 below 37 years old and 5 had no age reported. Ever since the *Echinococcus* control programme has been introduced in Greece, echinococcosis incidence has been decreased from 1.000 cases per year during the 80 s to 421 for the decade1998–2009 [4].

The aforementioned surveillance data though, might be misleading as long asymptomatic period is related to echinococcosis, and additionally the surveillance system in Greece suffers from underdiagnosis and underreporting [5].

To further investigate the epidemiology of echinococcosis, a study was designed to determine the seroprevalence of *Echinococcus granulosus* antibodies in the population of Thessaly, Central Greece, which is a predominantly agricultural region.

## Methods

During April to May 2009, a total of 542 left over blood samples were collected from public and private laboratories in the region of Thessaly and all of them were anonymous. Approval to use these samples was received by the Scientific Committee of the Post graduate Program in “Public Health and Environmental Hygiene”, University of Thessaly. After verbal consent was acquired, anonymous left over blood samples were collected and sent to the Department of Microbiology, General University Hospital of Larisa, Greece. For calculating the needed sample, we used as expected prevalence 1% (±0.7%) at a confidence level of 90%. The sample was stratified according to the population of the prefectures (Larissa, Trikala, Karditsa), residence (rural/urban), sex (male/female) and age group (0–14, 15–24, 25–34, 35–44, 45–54, 55–64, 65–74, >74). The left over sampling methodology implemented was based on the sampling methodology described by ESEN2 network (European Seroepidemiology Network) [6]. This way of sampling has the advantage of being of low cost and with sufficient representativeness. From each participating laboratory in the three prefectures we asked for specific number of samples fulfilling the inclusion criteria (age, sex, residence) according to the population of each prefecture. The samples were collected prospectively in three months period, while people were using the laboratory services for other purposes: checkups, other illness examinations etc.

An enzyme linked immunosorbent assay (Virion/Serion) was used to detect anti-Echinococcal IgG antibodies. The analysis was performed in the Department of Microbiology, General University Hospital of Larissa. Data were analyzed using the statistical package SPSS (v. 15). Fisher's exact test was used for qualitative data. Logistic regression analysis was used to control for confounders. Results were considered statistically significant when the p value was ≤0.05.

## Results

Out of 542 serum samples 6 (1.1%) were found positive for *Echinococcus granulosus* IgG antibodies. The descriptive characteristics of the samples in terms of gender, region, place of residence and age group are being presented in Table 1. Univariate statistical analysis was conducted with relation to gender, residence and age. As shown in Table 2, there was no statistically significant difference between men and women, while rural residence was found as a risk factor to seropositivity (RR = 7.60, 95% CI 0.89–64.64, p value 0.039). Finally in relation to the age group, 5 out of 6 positive samples belonged to the group 65–74 years. Multiple logistic regression analysis conducted, indicated that the most important factor affecting the results of this study was the age, with the age group of 65 and above to significantly have higher seropositivity (OR = 17.95, 95%CI 2.04–157.11, p value 0.009) (Table 3).

**Table 1:** Sample Descriptive Characteristics.

Characteristics	n/Total (N = 542) (%)
**Sex**	
*Male*	277 (51.1)
*Female*	265 (48.9)
**Area**	
*Karditsa*	122 (22.5)
*Larisa*	286 (52.8)
*Trikala*	134 (24.7)
**Residence**	
*Urban*	327 (60.3)
*Rural*	215 (39.7)
**Age group**	
*0–14*	86 (15.9)
*15–24*	70 (12.9)
*25–34*	75 (13.8)
*35–44*	73 (13.5)
*45–54*	69 (12.7)
*55–64*	67 (12.4)
*65–74*	66 (12.2)
*>74*	36 (6.6)

**Table 2:** Univariate Analysis.

Risk Factor	Sample value (> = 15)		
	N/Total (%)	RR(95% CI)	P-value*
**Sex**			0.364
*Male*	4/277 (1.4)	1.91	
*Female*	2/265 (0.8)	(0.35–10.36)	
**Residence**			**0.039**
Rural	5/215(2.3)	7.60	
*Urban*	1/327 (0.3)	(0.89–64.64)	
**Age Group**			**0.001**
*≥65*	5/102 (4.9)	21.57	
*<65*	1/440 (0.2)	(2.55–182.63)	

* **: Fisher's Exact test.**

**Table 3:** Multivariate Analysis.

Self-reported risk factor	Sample value (> = 15)	
	OR(95% CI)	P-value
**Sex**	1.70	0.551
*Male/Female*	(0.30–9.75)	
**Residence**	4,96	0.151
*Rural/Urban*	(0.56–44.15)	
**Age Group**	17,95	**0.009**
*≥65/<65*	(2.04–157.91)	

## Discussion

The seropositivity of *Echinococcus granulosus* in Central Greece was found 1.1% (0.5–2.5), results that could be considered in line to the surveillance data for Thessaly, for the decade 1998–2009. The multivariate analysis conducted in our study, revealed that the main affecting factor for the results was age, with age group of 65 and above to significantly associate with the seropositivity. This could be explained by the increased possibility to acquire antibodies against *Echinococcus granulosus* as age is progressing, without though necessarily developing the illness [3]. Moreover, it has been reported that only 10–20% of the diagnosed cases are under the age of 16 years [1]. The absence of cases and seropositive results in younger population may be also related to the reduction of the disease transmission, due to the control measures taken. The implementation of stricter regulations (E.C. Regulation 1774/2002) in relation to by products handling in the abattoirs has assisted this progress in *Echinococcus* control. Nowdays, the abattoir by products are being incinerated and by this way stray dogs cannot access them. As a result the biological cycle of *Echinococcus* is being restricted.

Another risk factor identified in our study, was the rural residence. Rural areas present an increased number of seropositive persons that is closely related to *Echinococcus* biological cycle. Greece has increased number of sheep and goat farms and the *Echinococcus* prevalence in livestock is 23–39.2% in sheep and 7.6–14.7% in goats [7]. Every herd owns sheepdogs that are not regularly dewormed and therefore it was expected to have higher prevalence in rural areas as reported by others [1].

Similar studies have been conducted in other European countries and the concluding remarks were in agreement with ours. In a study performed in Spain in 2003, anti Echinococcal antibody seroprevalence was found 3.4% and was significantly associated with age [8]. Another study conducted in Slovenia in 2008, concluded that the decrease of echinococcosis reported was mainly related to the control of *Echinococcus* in livestock [9].

According to the official data of the Hellenic Centre of Disease Control and Prevention [5], a decreasing trend in *Echinococcus* prevalence is being observed. Our study supports the above findings, alongside with a previous study in the same area, that determined ultrasonograhic prevalence of *Echinococcus* [10]. Moreover, similar results were found in a study in the island of Crete, that determined *Echinococcous* seroprevalence with counterimmunoelectrophoresis (CIE) [11].

Limitations of our study might have been the sampling and the method of antibody detection used. To facilitate the sample collection, we decided to use the methodology of left over blood samples, as used in similar seroprevalence studies [12]. Nevertheless, the sampling methodology implemented, could be considered as a convenient sample, stratified according to age, sex and residency of the total population in each prefecture. Major advantages of this sampling technique are the limited cost and the possibility of repeating the study using the same methodology to identify trends. A possible limitation of this sampling methodology is the lack of randomization, challenging the representativeness of the sample.

Regarding the method of antibody detection limitation, serum tests are not considered to be 100% sensitive and specific. Moreover the ELISA test specificity and selectivity relies mainly upon the kit used and more specifically on the antigen source used. A wide range from 31 to 96% for sensitivity and from 40–100% for specificity of ELISA kits has been reported [13], [14]. IgG antibodies production mainly depends on the number, the size, the topography and the hydatides cysts condition [1]. No production of antibodies has been also reported when small cysts, intact cysts and other highly calcified cysts exist [15]. Specificity issues on the other hand, might be as well complicated, when cross-reactions take place with other types of *Echinococcus* or in patients with cysticercosis [16]. Nonetheless, the ELISA test by detecting IgG antibodies shows previous contact with *Echinococcus granulosus*, without though confirming the illness.

Surveillance data and our study results converge that echinococcosis is being reduced in Greece, with older population to be affected mostly. These might be due to the disease transmission restriction, by the control measures being implemented. Efforts should be continued, in both animals and humans side, by increasing training campaigns and public awareness.
